# Exosomal transfer of bone marrow mesenchymal stem cell-derived miR-340 attenuates endometrial fibrosis

**DOI:** 10.1242/bio.039958

**Published:** 2019-03-19

**Authors:** Bang Xiao, Yiqing Zhu, Jinfeng Huang, Tiantian Wang, Fang Wang, Shuhan Sun

**Affiliations:** Department of Medical Genetics, Second Military Medical University, Shanghai 200433, China

**Keywords:** MicroRNA-340, Exosomes, Bone marrow mesenchymal stem cells, Endometrium injury

## Abstract

Bone marrow mesenchymal stem cells (BMSCs) have potential therapeutic benefits for the treatment of endometrial diseases and injury. BMSCs interact with uterus parenchymal cells by direct contact or indirect secretion of growth factors to promote functional recovery. In this study, we found that BMSC treatment in rats subjected to mechanical damage (MD) significantly increased microRNA-340 (miR-340) levels in the regenerated endometrium. Then we employed knockin and knockdown technologies to upregulate or downregulate the miR-340 level in BMSCs (miR-340^+^ BMSCs or miR-340^−^ BMSCs) and their corresponding exosomes, respectively, to test whether exosomes from BMSCs mediate miR-340 transfer. We found that the exosomes released from the primitive BMSCs or miR-340^+^ BMSCs but not miR-340^−^ BMSCs increased the miR-340 levels in primary cultured endometrial stromal cells (ESCs) compared with control. Further verification of this exosome-mediated intercellular communication was performed using exosomal inhibitor GW4869. Tagging exosomes with red fluorescent protein demonstrated that exosomes were released from BMSCs and transferred to adjacent ESCs. Compared with controls, rats receiving primitive BMSC treatment significantly improved functional recovery and downregulated collagen 1α1, α-SMA and transforming growth factor (TGF)-β1 at day 14 after MD. The outcomes were significantly enhanced by miR-340^+^ BMSC treatment, and were significantly weakened by miR-340^−^ BMSC treatment, compared with primitive BMSC treatment. *In vitro* studies reveal that miR-340 transferred from BMSCs suppresses the upregulated expression of fibrotic genes in ESCs induced by TGF-β1. These data suggest that the effective antifibrotic function of BMSCs is able to transfer miR-340 to ESCs by exosomes, and that enhancing the transfer of BMSC-derived miR-340 is an alternative modality in preventing intrauterine adhesion.

## Abstract (precis)

**Summary:** miR-340 in the exosomes released from BMSCs are transferred to endometrial cells, which regulate gene expression, repress endometrial fibrosis and promote functional recovery in rats subjected to mechanical damage.

## INTRODUCTION

Intrauterine adhesion (IUA) refers to the partial or complete atresia of the uterine cavity and/or cervical canal because of damage to the basal layer of the endometrium, resulting in amenorrhea, hypomenorrhea, infertility, recurrent pregnancy loss or abnormal placentation, including placenta previa and accreta (Conforti et al., 2013). The incidence of IUA varies between 2 and 22% of infertile women. Furthermore, 1.5% of women undergoing hysterosalpingography and 5% of women suffering recurrent miscarriages present with IUA (Khan and Goldberg, 2018). At present, we use intrauterine devices with high doses of estrogen to prevent IUA and promote endometrium regeneration after surgical synechiotomy in clinical settings (Song et al., 2016). This method may help alleviate the some basic symptoms, but re-adhesion often occurs.

Bone marrow mesenchymal stem cells (BMSCs) have potential therapeutic benefits in many diseases, including endometrial diseases and injury (Li et al., 2018; Travnickova and Bacakova, 2018). However, it is unknown how BMSCs communicate with uterus parenchymal cells to promote functional recovery.

A wide range of cell types secrete exosomes, which are membrane vesicles sized 40–100 nm in diameter (Bu et al., 2018). RNA molecules including messenger RNA (mRNA) and microRNA (miRNA) contained in these exosomes can be transferred between cells to influence the protein production of recipient cells (Zomer et al., 2010). Increasing evidence demonstrates that exosomes play a vital role in cellular communication (Pegtel et al., 2010).

In eukaryotic cells, miRNAs constitute an important regulatory system (Ambros, 2001; Ke et al., 2003). There are over 1000 miRNAs encoded from human genome (Friedman et al., 2009). These miRNAs are abundant and involved in most biological processes through targeting nearly 60% of genes in many cell types (Lewis et al., 2005). Recently, the role of miRNAs at various stages of endometrium development has been elucidated (Paul et al., 2018). Consistent with the hypothesis that miRNAs have vital roles in the gene regulatory networks involved in the dynamic and cyclical redevelopment of endometrium, numerous miRNAs are expressed in spatially and temporally controlled manners in the endometrium (Paul et al., 2018; Ferlita et al., 2018).

We hypothesized that BMSCs communicate with parenchymal cells through transferring miRNA between BMSCs and parenchymal cells by exosomes, which may contribute to the improvement of endometrial function after IUA via regulating specific gene expression.

In this study, we focused on miR-340 in the endometrium after mechanical damage (MD) and BMSC treatment. Since ESCs are the essential cells for the functional recovery after IUA and the major endogenous repair mediator in uterus, in this study we used primary cultures of ESCs as the representative parenchymal cells. *In vitro*, we investigated whether the miR-340 is transferred to parenchymal cells via exosomes generated by BMSCs. *In vivo*, we upregulated or downregulated the miR-340 level in BMSCs (miR-340^+^ BMSCs or miR-340^−^ BMSCs) and their corresponding exosomes, respectively, and then administered these BMSCs to rat uterus subjected to MD to test whether the exosomes mediate miR-340 transfer to endometrial cells to promote functional recovery.

## RESULTS

### BMSC administration significantly increases miR-340 level after injury

**Fig. 1.**
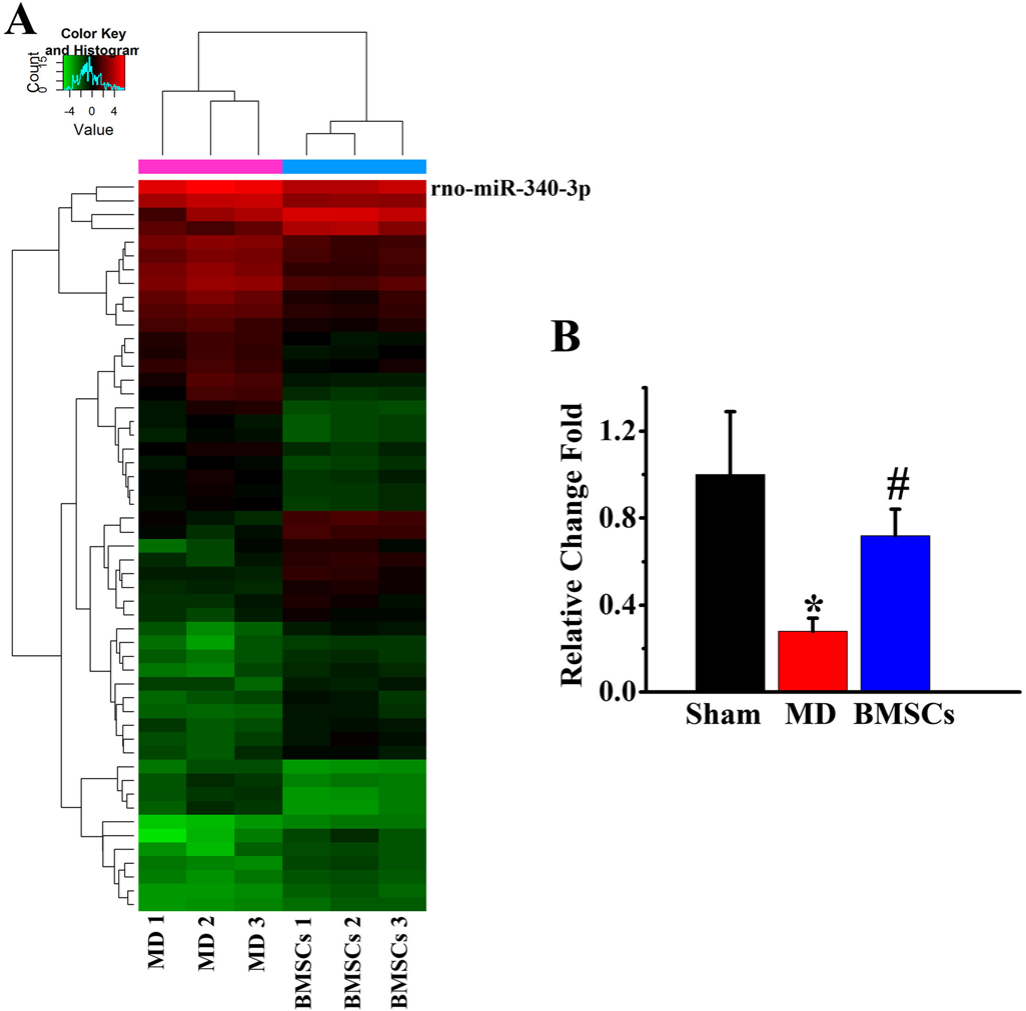
**BMSC administration increases the miR-340 after mechanical damage (MD).** (A) The heat map shows relative expression of small RNAs in endometrial tissues of rats in MD group and BMSC administration group. (B) Real-time reverse-transcribed PCR assay shows miR-340 significantly decreased in the endometrial tissues of rats subjected to MD compared with sham rats, and BMSC administration significantly increased the miR-340 level in the MD rat endometrium. *P<0.05 compared with sham. ^#^*P<*0.05 compared with MD (*n*=6 per group).

We extracted the total RNA from the normal and MD rat endometrium with or without BMSC administration. A supervised clustering analysis showed distinct miRNA transcriptome signatures in endometrium of rats in MD control group and BMSC administration group. Among the miRNAs and that were identified, miR-340 was the most abundant (Fig. 1A). Real-time PCR results showed that compared to normal rat endometrium, miR-340 was significantly decreased in the endometrium of rats subjected to MD and BMSC administration significantly increased the miR-340 level in MD rat endometrium compared to the MD control (Fig. 1B).

### Overexpressed miR-340 in BMSCs and corresponding exosomes

**Fig. 2.**
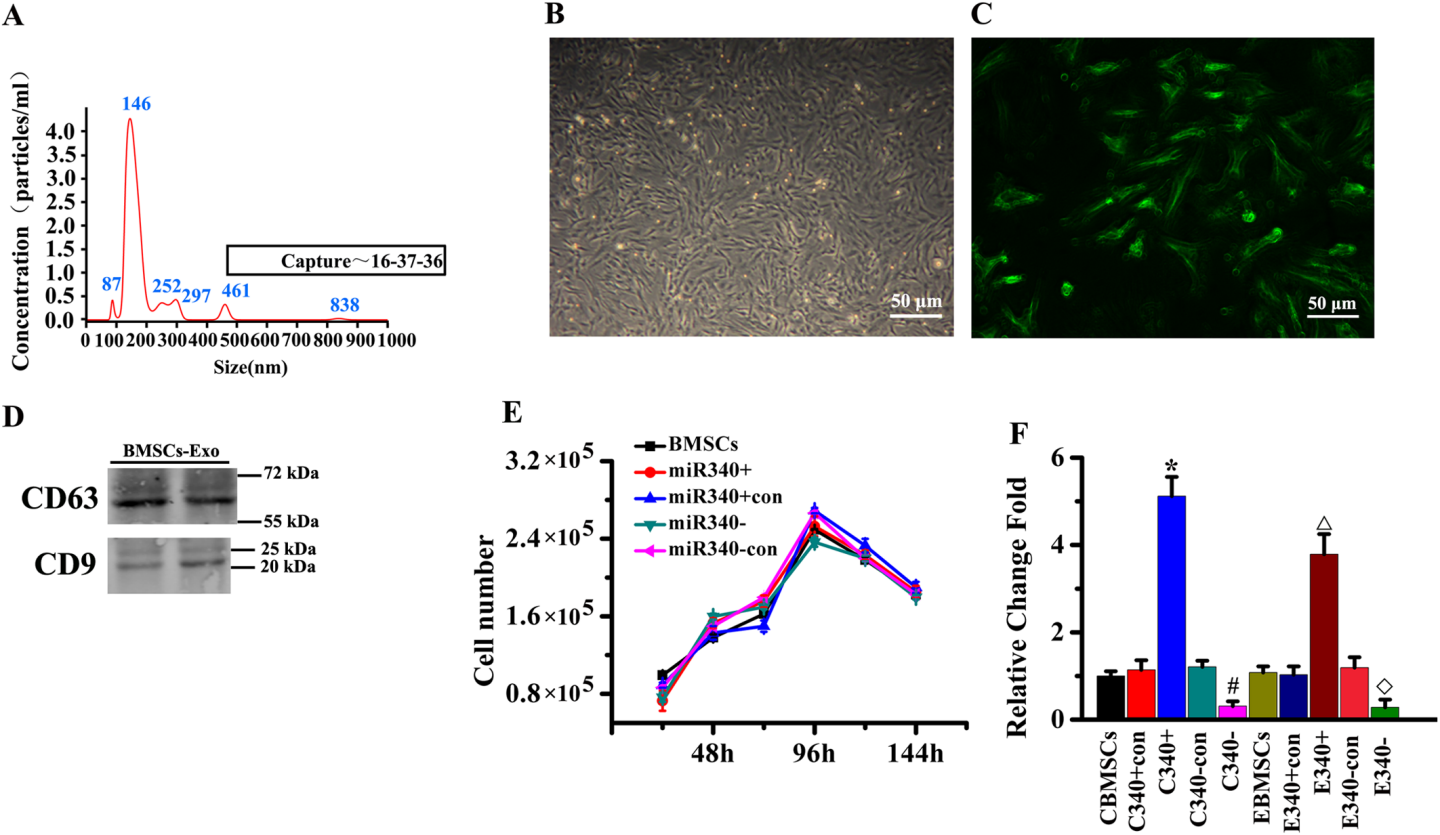
**The miR-340 expression in BMSCs and their generated exosomes.** (A) Nanoparticle analysis of exosomes derived from BMSCs. The mean diameter was 182 nm. (B) The morphological characteristics of the BMSCs of passage 5 after transfection under the optical microscope. (C) Fluorescence microscopy for GFP^+^ expression showed the transfection efficiency of BMSCs. (D) Immunoblotting for CD63 and CD9 in exosomes. (E) Growth curve analysis shows that miR-340^+^ BMSCs and miR-340^−^ BMSCs and their corresponding control BMSCs exhibit similar cell proliferation character. (F) RT-PCR data show the miR-340 levels in miR-340^+^ BMSCs and their exosomes were significantly increased compared with those in control; however, miR-340^−^ BMSCs exhibited significantly decreased miR-340 expression level in cells and exosomes compared with those in control. *P<0.05, compared with C340+con; ^#^*P*<0.05 compared with C340−con; ^△^*P*<0.05 compared with E340+con; ^◇^*P*<0.05 compared with E340−con (*n*=6 per group).

In order to determine whether the miR-340 is contained in exosomes released from BMSCs, we used the standard exosome isolation method of ultracentrifugation, nanoparticle analysis of exosomes derived from BMSCs. The mean diameter was 182 nm (Fig. 2A). Western blot analysis showed that exosomes from BMSCs expressed the exosomal markers CD63 and CD9 (Fig. 2B). We infected BMSCs with LentimiRa-GFP-rno-mir-340 lentivirus (miR-340^+^ BMSCs), GFP Blank miRNA lentivirus (miR-340^+CON^ BMSCs), anti-miR-340 lentivirus (miR-340^−^ BMSCs) and pGreenPuro Scramble Hairpin Control lentivirus (miR-340^−CON^ BMSCs), respectively. The transfection of miR-340^+^ BMSCs and miR-340^−^ BMSCs as well as their corresponding control miR-340^+CON^ BMSCs and miR-340^−CON^ BMSCs showed a high efficiency of GFP expression by immunofluorescence microscopy (Fig. 2C). To test whether miR-340 knockin or knockdown affects the characteristics of BMSCs, we performed cell growth curve analysis. Our results indicated that there were no significant differences in proliferation among the four types of cells (Fig. 2D). Our data also show that the expression of miR-340 in miR-340^+^BMSCs and in their exosomes were significantly higher than those in their corresponding control, and the expression of miR-340 in miR-340^−^ BMSCs and in their exosomes were significantly lower than those in their corresponding control (Fig. 2E). These results revealed that miR-340^+^ BMSCs or miR340^−^ BMSCs successfully increase or decrease miR-340 levels in the BMSCs and their exosomes, respectively.

### Exosomal miR-340 of BMSCs is transferred to ESCs

**Fig. 3.**
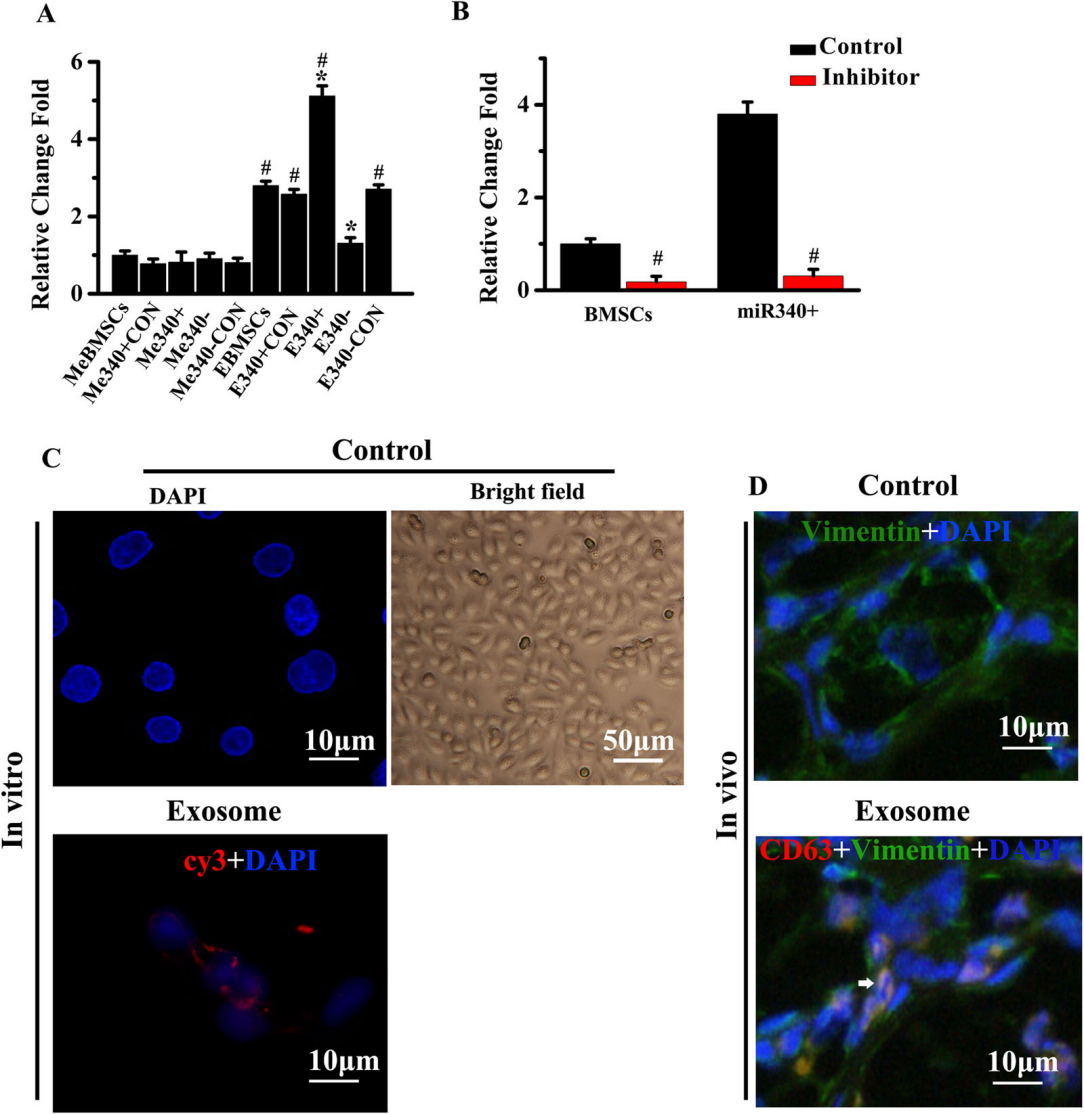
**BMSCs exosomes containing miR-340 are transferred to ESCs.** (A) Real-time reverse-transcribed PCR revealed that compared with exosome-deprived media, exosomes collected from naive BMSCs or BMSCs transfected with miR-340^+CON^, miR-340^+^ and miR-340^−CON^ increased the miR-340 level in ESCs. (B) The addition of an exosome inhibitor, GW4869, was confirmed to inhibit the elevated expression of miR-340 in ESCs when co-cultured with naive BMSCs or miR-340^+^ BMSCs. (C) Exosomes were isolated from conditioned media of BMSCs transfected with cy3-labeled miR-340 and added to ESCs cultures. ESCs were fixed and the nuclei were stained with 4,6-diamidino-2-phenylindole (DAPI) blue. An SP5 confocal microscope was used to detect the signals in ESCs. (D) Exosomes of BMSCs in the endometrium are taken up by adjacent ESCs (arrow). *P<0.05 compared with EBMSCs in A; ^#^*P*<0.05 compared with MeBMSCs in A, compared with control in B (*n*=6 per group).

To explore whether the miR-340 is transferred from BMSCs to uterus parenchymal cells, we collected the exosomes from naive BMSCs or BMSCs transfected with miR-340^+CON^, miR-340^+^, miR-340^−^ and miR-340^−CON^ BMSCs and added them to primary culture ESCs. Real-time PCR results show that compared to exosomes deprived media, exosomes collected from naive BMSCs or BMSCs transfected with miR-340^+CON^, miR-340^+^ and miR-340^−CON^ increased the miR-340 level in ESCs (Fig. 3A). Compared with ESCs treated with exosome-enriched fractions from naive BMSCs, miR-340 levels were significantly increased in ESCs treated with exosome-enriched fractions from miR-340^+^ BMSCs while significantly decreased after treatment of exosomes from miR-340^−^ BMSCs. To further verify that miR-340 is transferred from exosome-enriched fractions of BMSCs, the exosome inhibitor, GW4869, was used to treat the miR-340^+^ BMSCs or naive BMSCs co-cultured ESCs. Data showed that the miR-340 levels were significantly decreased in the miR-340^+^ BMSCs or naive BMSCs co-cultured ESCs after addition of GW4869 (Fig. 3B). To delineate the transfer of miR-340 mediated by exosomes, we isolated exosomes from conditioned media collected from BMSCs transfected with cy3-tagged miR-340 and then added them to ESCs. Fluorescently labeled signals (arrowheads) were found in the ESCs incubated with these exosomes (Fig. 3C). To determine whether the exosomes mediate cell–cell communication between BMSCs and endometrial cells *in vivo*, we transfected BMSCs with the plasmid containing CD63-dsRedgene and injected these CD63-dsRed-BMSCs into rats subjected to MD. Considering CD63 is a common marker of exosomes, we visualized CD63-dsRed in exosomes using a confocal microscope. As shown in Fig. 3D, exosomes released from BMSCs were detected in adjacent ESCs (arrowheads). Taken together, these results confirm that miR-340 was transferred from BMSCs to ESCs via exosomes.

### Exosomal transfer of miR-340 mediates BMSC-induced functional recovery

**Fig. 4.**
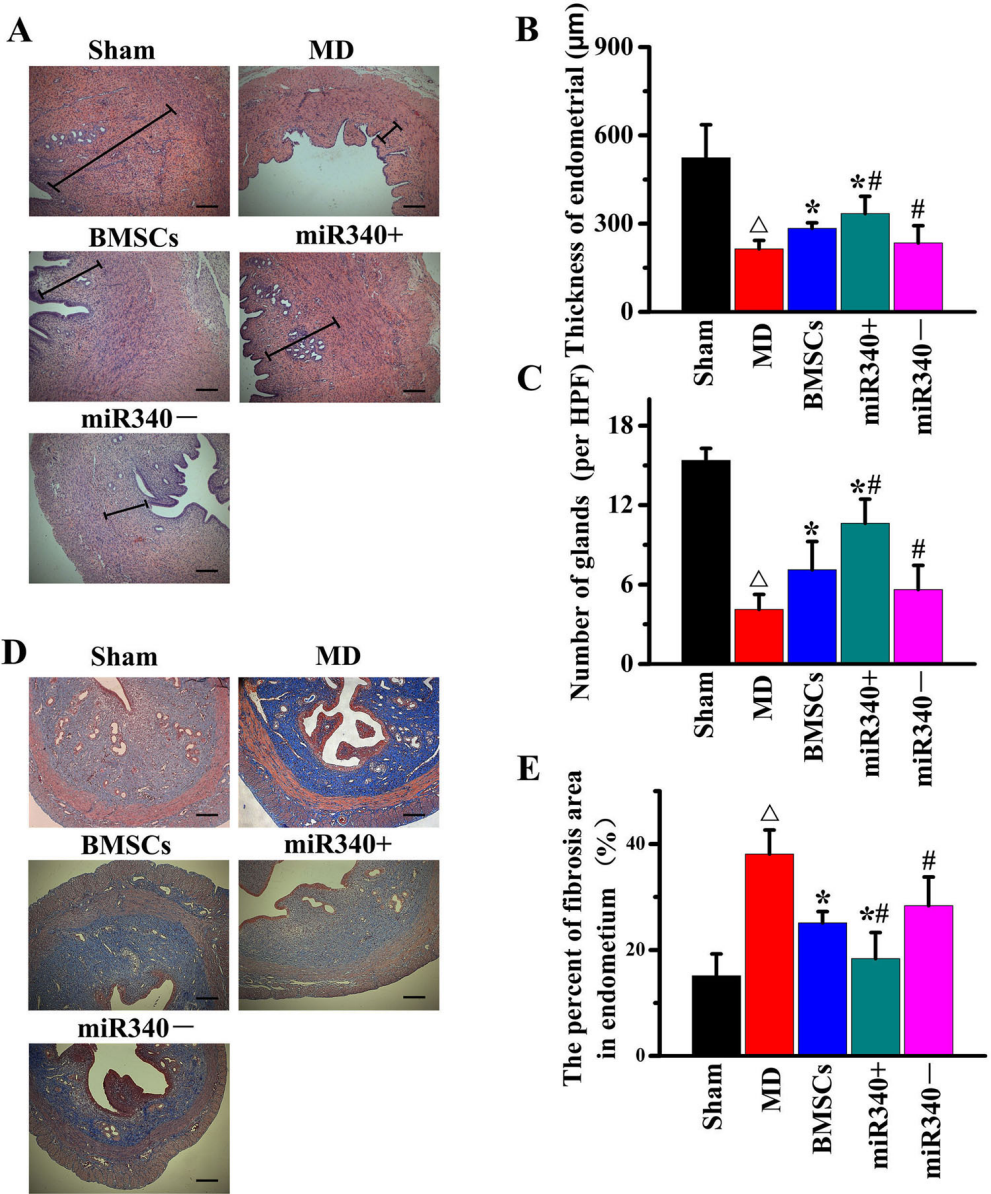
**miR-340 increases functional regeneration of injured rat uterus.** (A) Representative image shows Hematoxylin and Eosin (HE) staining in a rat endometrium section. (B,C) Analysis data show that thickness of endometrium (B) and number of glands (C) was significantly increased after naive BMSC treatment compared with PBS treatment at day 14 after MD. miR-340^+^ BMSC treatment significantly increased while miR-340^−^ BMSC treatment significantly decreased the thickness of the endometrium and number of glands at day 14 after MD compared with naive BMSC treatment. (D) Masson’s Trichrome staining performed on adjacent frozen endometrium sections to detect the fibrosis. (E) Compared with PBS treatment, the positive staining significantly decreased at day 14 after MD in the endometrium after naive BMSC treatment. Compared with naive BMSC treatment, miR-340^+^ BMSC treatment significantly decreased, while miR-340^−^ BMSC treatment significantly increased the positive staining area at day 14 after MD. ^△^*P*<0.05 compared with sham; *P<0.05 compared with MD control; ^#^*P*<0.05 compared with naive BMSCs. Scale bars: 250 μm, mean±s.e.m., *n*=6/group.

24 h after MD, we injected naive BMSCs, PBS and four types of modified BMSCs into rats (3–5×10^6^/rats in 200 μl PBS) via the caudal vein (*n*=10/group). Hematoxylin and Eosin (HE) staining was performed prior to the treatment and at day 14 (Fig. 4A). Compared with PBS treatment, naive BMSCs, miR-340^+CON^ BMSCs and miR-340^−CON^ BMSCs significantly increased the thickness of endometrium (Fig. 4B) and number of glands (Fig. 4C). Compared with naive BMSC treatment, miR-340^+^BMSC treatment significantly increased while miR-340^−^ BMSC treatment significantly decreased the thickness of endometrium and number of glands. We also performed Masson's Trichrome staining on adjacent frozen endometrium sections to detect the fibrosis (Fig. 4D). Compared with PBS treatment, the positive staining significantly decreased in the endometrium after naive BMSC treatment. Compared with naive BMSC treatment, miR-340^+^ BMSC treatment significantly decreased while miR-340^−^ BMSC treatment significantly increased the positive staining area at day 14 after MD (Fig. 4E). These results indicate that increasing the expression of miR-340 in BMSCs and their released exosomes enhances functional regeneration of injured rat uterus.

### BMSCs regulate fibrotic gene expression in ESCs by transfer of miR-340 via exosomes

**Fig. 5.**
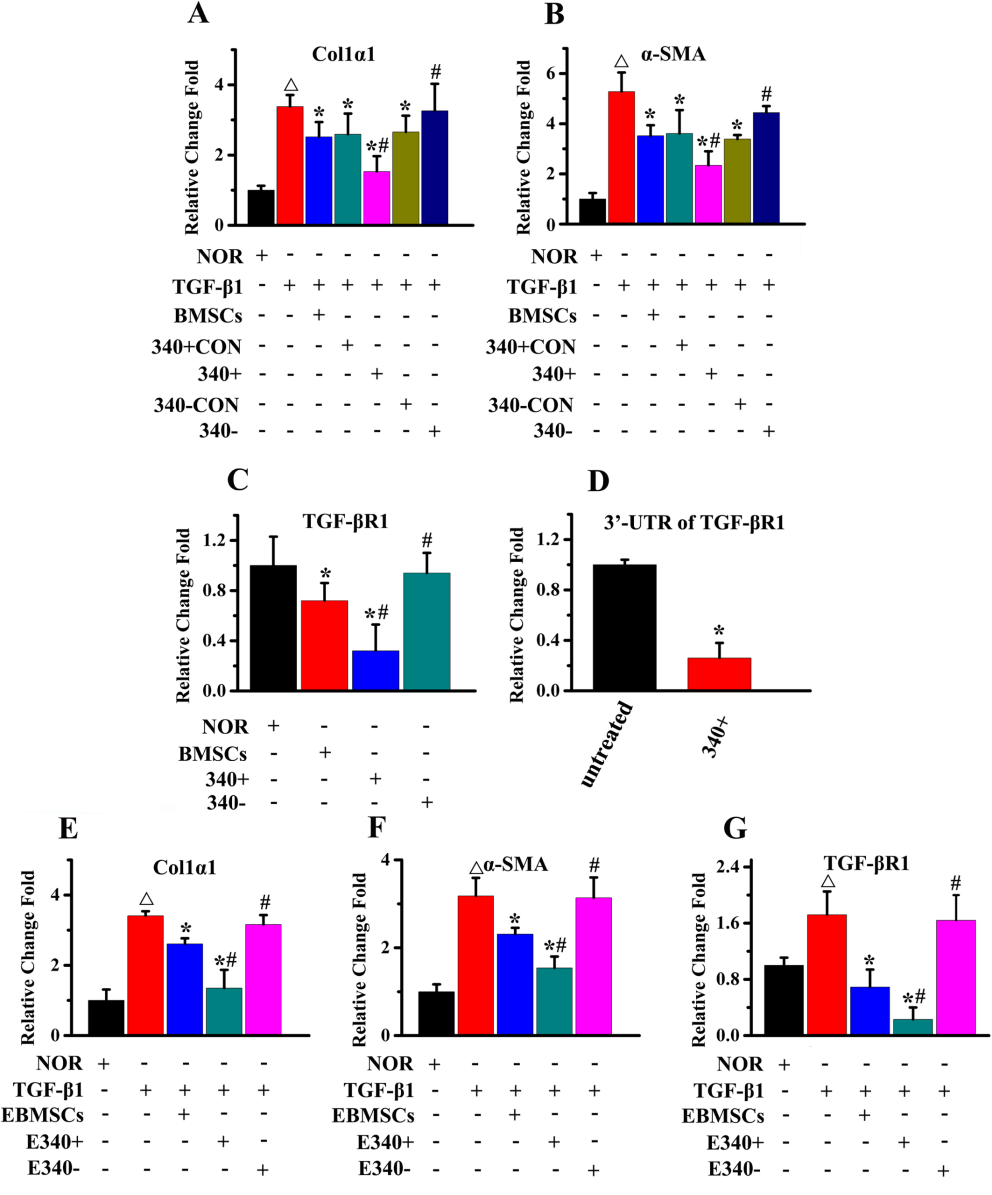
**miR-340^+^ BMSCs regulate fibrotic gene expression in ESCs through exosomes.** (A,B) The upregulated expression of Col1α1 (A) and α-SMA (B) in ESCs, stimulated by the addition of TGF-β1, was significantly reduced following co-culture with miR-340^+^ BMSCs and to a lesser extent with naive BMSCs, while miR-340^−^ BMSC treatment significantly increased the expression of collagen 1α1 and α-SMA compared with naive BMSC treatment. (C) The expression of TGF-βR1 was significantly reduced following co-culture with miR-340^+^ BMSCs and to a lesser extent with naive BMSCs, while miR-340^−^ BMSC treatment significantly increased the TGF-βR1 expression compared with naive BMSC treatment. (D) The co-culture of miR-340^+^ BMSCs reduced the 3′UTR TGF-βR1 expression in ESCs. (E–G) ESCs were treated with exosomes isolated from the conditioned media of naive BMSCs, miR-340^+^ BMSCs and miR-340^−^ BMSCs culture for 72 h in the presence of TGF-β1. The addition of the isolated exosomes reduced the upregulated expression of (E) Col1α, (F) α-SMA and (G) TGF-βR1. Results are expressed as the mean±s.e.m. ^△^*P*<0.05 compared with NOR in A,B,E,F,G; *P<0.05 compared with TGF-β1 in A,B,E,F,G, compared with NOR in C and compared with untreated in D; ^#^*P*<0.05 compared with BMSCs in A,B,C and compared with EBMSCs in E,F,G.

To determine the efficacy of miR-340 delivered from BMSCs, we performed qPCR assay to detect key genes involved in endometrial fibrosis in ESCs in response to TGF-β1. ESCs were co-cultured with naive BMSCs and four types of modified BMSCs using the indirect transwell with or without the addition of TGF-β1. The upregulated expression of collagen 1α1 (Fig. 5A) and α-SMA (Fig. 5B) in ESCs, stimulated by the addition of TGF-β1, were significantly reduced following co-culture with miR-340^+^ BMSCs and to a lesser extent with naive BMSCs, while miR-340^−^ BMSC treatment significantly increased the expression of collagen 1α1 and α-SMA compared with naive BMSC treatment. The gene expression of TGF-βR1 was determined in ESCs co-cultured with or without naive BMSCs, miR-340^+^ BMSCs and miR-340^−^ BMSCs, revealing a reduction of TGF-βR1 by naive BMSCs, miR-340^+^ BMSCs but not miR-340^−^ BMSCs co-culture treatment (Fig. 5C). miRNAs are known to regulate target mRNA by base pairing to partially complementary sites in 3′UTRs preventing translation, so we transfected ESCs with 3′UTR of TGF-βR1. The luciferase reporter assay indicated that miR-340^+^ BMSCs repressed luciferase activity for the 3′UTR wild-type constructs in ESCs (Fig. 5D) as compared with co-culture with ESCs alone. To further verify the pivotal role of exosomes as a fibrotic regulator, ESCs were treated with exosomes isolated from naive BMSCs, miR-340^+^ BMSCs or miR-340^−^ BMSCs conditioned medium in the presence of TGF-β1 after 72 h of culture. The upregulated expression of collagen 1α1 (Fig. 5E), α-SMA (Fig. 5F) and TGF-βR1 (Fig. 5G) in ESCs, stimulated by the addition of TGF-β1, was significantly reduced following addition of exosomes from miR-340^+^ BMSCs and to a lesser extent with naive BMSCs, while exosomes from miR-340^−^ BMSC treatment significantly increased the expression of collagen 1α1, α-SMA and TGF-βR1 compared with exosomes from naive BMSC treatment.

### miR-340 regulates fibrotic gene expression in the endometrium of rat subjected to MD

**Fig. 6.**
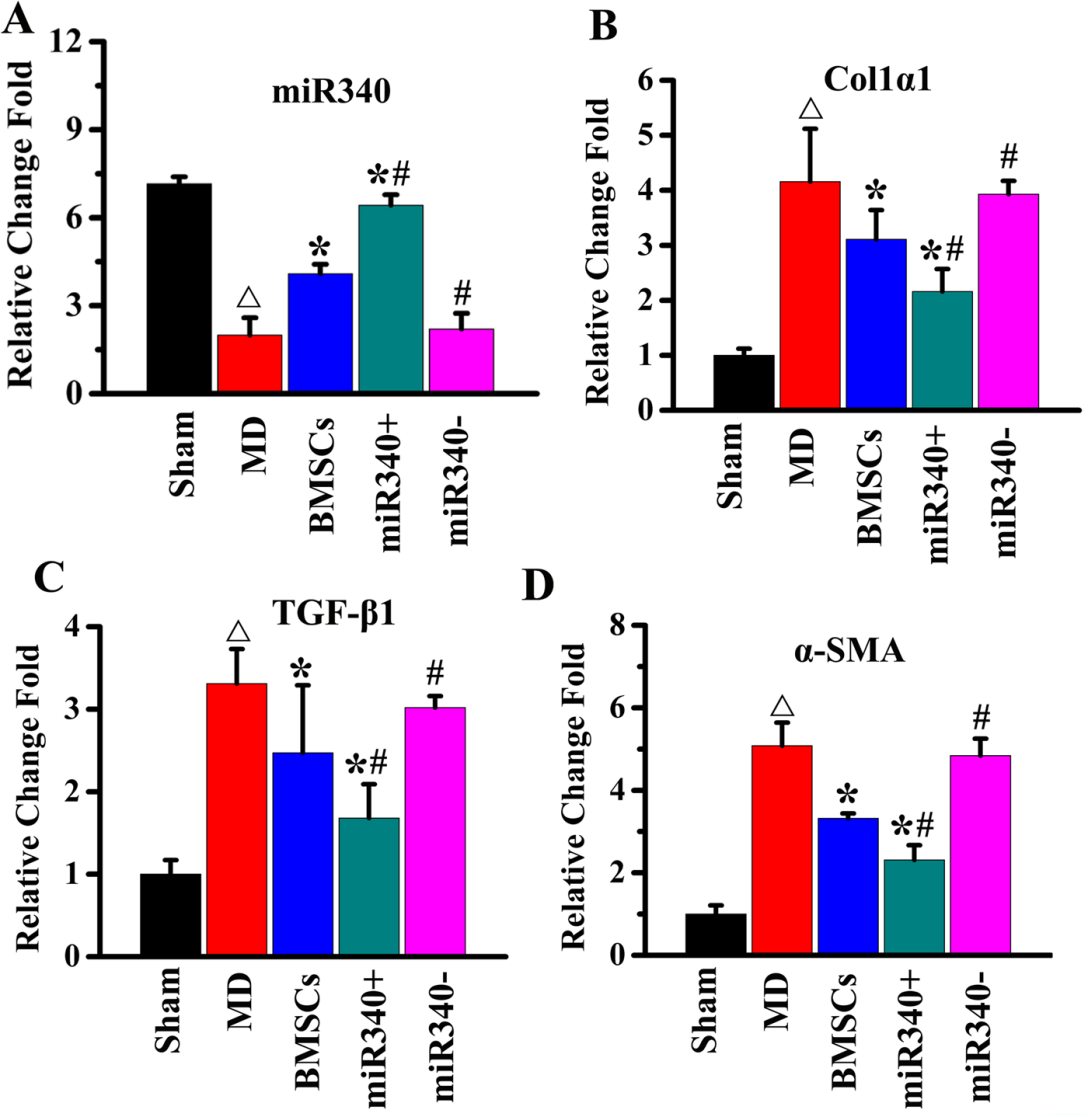
**miR-340 regulates fibrotic gene expression in the endometrium.** Real-time reverse-transcribed PCR assay showed the expression of (A) miR-340 significantly decreased while (B) col1α1, (C) col4α1 and (D) α-SMA expression significantly increased at day 14 after MD compared with sham rats. The administration of naive BMSCs significantly increased miR-340 expression and decreased the col1α1,col4α1, α-SMA expression. miR-340^+^ BMSC treatment further significantly increased miR-340 expression and decreased the col1α1, col4α1 and α-SMA expression compared with naive BMSC treatment, while miR-340^−^ BMSC treatment decreased the miR-340 expression and sustained the col1α1,col4α1 and α-SMA expression at a significantly elevated level compared with naive BMSC treatment. ^△^*P*<0.05 compared with sham; *P<0.05 compared with MD; ^#^*P*<0.05 compared with BMSCs. Mean±s.e., *n*=6/group.

qPCR was employed to measure the relative expression of miR-340, collagen1α1, TGF-β1 and α-SMA following 14 days of treatment with/without naive BMSCs or four types of modified BMSCs in MD rat. Our data show that the expression of miR-340 (Fig. 6A) significantly decreased while col1α1 (Fig. 6B), TGF-β1 (Fig. 6C) and α-SMA (Fig. 6D) expression significantly increased at day 14 after MD compared with sham rats. The administration of naive BMSCs significantly increased miR-340 expression and decreased the col1α1, TGF-β1 and α-SMA expression at day 14 after MD. miR-340^+^ BMSC treatment further significantly increased miR-340 expression and decreased the col1α1, TGF-β1 and α-SMA expression compared with naive BMSC treatment, while miR-340^−^BMSC treatment decreased the miR-340 expression and sustained the col1α1, TGF-β1 and α-SMA expression at a significantly elevated level compared with naive BMSC treatment.

## DISCUSSION

BMSC transplantation has potential therapeutic benefits for the treatment of endometrium injury or preventing diseases, such as intrauterine adhesion (Wang et al., 2016; Ding et al., 2014). BMSCs contribute to endometrium functional recovery by interacting with uterus parenchymal cells and promote the proliferation of ESCs (Zhang et al., 2018). This interaction includes direct cell–cell communication or indirect pattern being mediated by the secretion of factors by BMSCs (Gan et al., 2017). After injury, only a quite small percentage of injected BMSCs could enter the endometrium because of the extremely low recruitment efficiency. The mechanisms underlying how the relatively few administrated BMSCs resident in endometrium make such a significant contribution to functional recovery after injury are not fully uncovered. Exosomes act as mediators of cell–cell communication and are carriers for small regulatory molecule, such as miRNA, delivery (Yang et al., 2011). Recently, exosomes are being considered to be used as biomarkers or potential therapeutic tools in cancer (Bu et al., 2018). Some previous studies on the nervous system suggested that miRNA transfer from MSCs to parenchymal cells mediated by exosomes modulated the neural cell gene expression and protein production which promote neurite outgrowth (Xin et al., 2012). In the current study, we demonstrated that miR-340 level was substantially decreased in rat endometrium after MD, and BMSC administration significantly increased the miR-340 level, suggesting that miR-340 plays a role in modulating the endometrium recovery process facilitated by BMSCs.

Then we explored whether the miR-340 is transferred from BMSCs to uterus parenchymal cells. In *in vitro* studies, we found that that exosomes collected from naive BMSCs or BMSCs transfected with miR-340^+CON^, miR-340^+^, miR-340^−CON^, but not exosomes deprived media, increased the miR-340 level in ESCs. This process was further verified using an exosomes inhibitor, GW4869, which blocked the transfer of miR-340 from miR-340^+^ BMSCs or naive BMSCs to ESCs in a co-cultured system. In *in vivo* studies, exosomes released from BMSCs were found in adjacent ESCs through detecting a common marker of exosomes, CD63, which is tagged by dsRed in CD63-dsRed-BMSC constructs injected into rat subjected to MD. These data suggest that the exosomes mediate the miR-340 transfer from BMSCs to ESCs.

MiRNAs play key roles in development and regeneration of the endometrium which is immensely dynamic and cyclically redeveloped (Jimenez et al., 2016; Lam et al., 2012). In the injured endometrium, the scar, which is composed of excessive extracellular matrix (ECM) and myofibroblasts, represents a major impediment to regeneration (Salazar et al., 2017). TGF-β1 plays an important role in promoting fibrosis by mediating ECM production and myofibroblast transition (Tang et al., 2018; Krummel et al., 1988). In this study, our data suggests that TGF-β1 induces myofibroblast transdifferentiation of ESCs, verified by the increased expression of α-SMA, which plays a vital role in the progress of endometrium fibrosis and increases collagen1α1 level. The phenotypic transformation of ESCs led to hypertrophy, accompanied with a significantly increased secretion of ECM components or inflammatory factors, which results in a vicious circle that promotes endometrium fibrosis (Gressner and Weiskirchen, 2006). Notably, the present study demonstrates that exosomal transfer of BMSC-derived miR-340 increases the expression of miR-340 in ESCs and is capable of inhibiting the TGF-β1-induced expression of collagen1α1 and α-SMA to prevent endometrium fibrosis *in vitro*. Meanwhile, our *in vivo* studies also showed that administration of naive BMSCs reduces endometrium damage and collagen accumulation, and miR-340^+^ BMSC therapy further enhances the protective benefits, while miR-340^−^ BMSC treatment weakens the protective benefits. These results indicate that miR-340 represses the endometrium fibrosis. In addition, our results also show that naive BMSCs and miR-340^+^ BMSCs can repress the 3′UTR expression of TGFβ-R1, suggesting TGFβ-R1 is a target of miR-340.

Endometrium injury influenced the production and composition of BMSC-released exosomes that mediate the communication of BMSCs and endometrial cells promoting the anti-fibrosis effect, which may enhance functional recovery. Recently, one of the major challenges for clinical gene therapy applications is vehicles for diffuse delivery to the uterus, which may be conquered by using exosomes as a delivery vehicle. In addition, allogeneic BMSCs could escape immune system surveillance and survive in the uterus due to their ability to suppress T-cell-mediated responses for tissue rejection (Di Nicola et al., 2002; Li et al., 2006). Therefore, BMSCs that can provide a source of exosomes are an ideal cell source of exosomes for functional molecule delivery. We expect that application of exogenous BMSC-released exosome delivery of miR-340 or other beneficial miRNAs will further promote functional recovery to prevent intrauterine adhesion – also known as Asherman syndrome – after injury as compared with naive BMSC treatment.

### Conclusion

miR-340 in the exosomes released from BMSCs are transferred to endometrial cells, which regulate gene expression, repress endometrial fibrosis and promote functional recovery in rats subjected to MD.

## MATERIALS AND METHODS

All animal protocols were approved by the Institutional Ethics Committee of the Second Military Medical University, China, and were consistent with current regulations [GB14925-2001: Laboratory Animal Requirements of Environment and Housing Facilities (Chinese version)].

### MD model

Adult female Sprague-Dawley rats (200–220 g) purchased from SLAC Laboratory Animal Co., Ltd. (Shanghai, China) were subjected to MD in accordance with the method reported previously (Xu et al., 2017). Briefly, vaginal smears were taken daily and rats with four continuous 4-day estrus cycles were subjected to MD. Rats were anesthetized via lumbar injection of 3% mebumalnatrium (1.3 ml/kg), and were cut out in a low midline belly to expose the uterus. The uterus was incised a 1.0-cm longitude in the lowermost one-third of the joining between the middle and distal and then were scraped until its wall became coarse and with apparent endometrium distension.

### BMSC cultures and construction of stable miR-340 knocked in or knocked down BMSCs

Bone marrow from the rats (3 weeks old) was mechanically dissociated, and the bone marrow was collected using low glucose Dulbecco's Modified Eagle's Medium (LG-DMEM; Sigma) without serum. The bone marrow was then transferred in the centrifuge tubes (BD Biosciences) on the ficoll gradient (GE Healthcare Bio-Sciences). Proportion of ficoll and bone marrow was 1:3; the tubes were centrifuged at 300 ***g*** for 20 min. After centrifugation, we collected cells at a whitish ring and transferred them to a new centrifugation tubes and then washed them twice with LG-DMEM containing 10% of fetal bovine serum (FBS; Hyclone). Cells were cultivated in LG-DMEM with 10% fetal bovine serum, 100 U/ml penicillin (Sigma) and streptomycin (Sigma) at 5% CO_2_ and 37°C as previously optimized. Three days later, cells that tightly adhered to the cell culture dish were considered P0 BMSCs. Three to four passages (P3-4) of cells were used for lentivirus infection. BMSCs were passaged or collected for injection when they achieved 80%–90% confluence. Conventional culture medium was replaced with an exosome-free FBS (Hyclone) medium when the cells reached 60%–80% confluence to isolate the exosome. The media were collected for centrifugation after an additional 24 h of culture.

We packaged the lentiviruses containing the vectors of LentimiRa-GFP-rno-miR-340 Vector (LentiLab, pre-miR-340 inserted for miR-340 knockin), pLenti-III-miR-GFP Control Vector (LentiLab, vector for miR-340 knockin control), miRZip-340 anti-miR-340 microRNA construct (LentiLab, miR-340 inhibitor inserted for miR-340 knockdown) and pGreenPuro Scramble Hairpin Control Construct (LentiLab, vector for miR-340 knockdown control), respectively, according to the manufacturer's protocol. Then we generated miR-340 knocked in or knocked down BMSCs by infecting the primary cultured BMSCs with these lentiviruses. We monitored the infection efficiency and selected stable cell lines using the green fluorescent protein (GFP) and puromycin, respectively. We identified the four stable BMSC cell lines as: miR-340^+^ BMSCs, miR-340^+CON^ BMSCs, miR-340^−^ BMSCs and miR-340^−CON^ BMSCs, respectively.

### BMSC administration

At 24 h post injury of endometrium, rats randomly selected (*n*=15/group, *n*=90 in total) received naive BMSCs, miR-340^+^ BMSCs, miR-340^+CON^ BMSCs, miR-340^−^ BMSCs and miR-340^−CON^ BMSCs or vehicle administration. We injected approximately 4×10^6^ BMSCs in 500 μl phosphate-buffered saline or PBS alone via the tail vein slowly into each rat. We euthanized all the rats 14 days after MD. To measure the corresponding miR-340 level in the endometrium, endometrium samples were acquired immediately prior to euthanasia. At the end of the experiment, rats were excessively anesthetized via lumbar injection of 3% mebumalnatrium (3.9 ml/kg). Some rat endometriums were snap frozen in liquid nitrogen and stored at −80°C and some were fixed with 4% paraformaldehyde. A series of 50 mg frozen tissues were taken for molecular studies (western blot and RT-PCR) and 8 μm-thick fixed tissue was taken for histochemistry and immunostaining.

### Exosome isolation

We isolated exosomes from the condition medium (CM) of BMSCs with a modified differential ultracentrifugation method as previously reported (Au Yeung et al., 2016). Briefly, we removed contaminating cells through centrifuging the CM at 300× ***g*** for 10 min and then removed larger microvesicles (MVs) by centrifuging at 20,000× ***g*** for 30 min. Then we transferred the resulting supernatants to fresh tubes and filtered it through 0.8 μm filter GVS Maine Poretics, PCTE Filter Membranes (Thermo Fisher Scientific). We pelleted the enriched exosomes by centrifuging the filtered samples at 110,000× ***g*** for 2 h (Beckman). Then we re-suspended pellets in PBS and centrifuged them at 100,000× ***g*** for another 1 h. We re-suspended the exosome pellets in 50∼100 μl of PBS and stored at −80°C for further use.

### MiRNA assay

We lysed the samples isolated the total RNA using the Qiazol reagents and miRNeasy Mini kit (Qiagen), respectively to measure miR-340 in exosomes from cultured cells. We reverse transcribed the miRNAs using the miRNA Reverse Transcription kit (Applied Biosystems) and then performed PCR amplification with the TaqMan miRNA assay kit (Applied Biosystems) to detect the miR-340 level according to the manufacturer's instructions. U6 snRNA was used as an internal control.

### *In vitro* detection of miR340 transfer

For the exosome treatment experiment, BMSCs were transfected with 10 nM red fluorescent cy3-labeled miR340 for 24 h. We washed the cells with PBS and incubated them using freshly prepared complete medium containing exosome-free FBS for 48 h. We collected the BMSC-conditioned medium and isolated exosomes from the conditioned medium by differential centrifugation according to the procedure mentioned above. The pellet was suspended in exosome-free medium and used to treat ESCs grown on cover slips. After 24 h, we fixed the ESCs with 4% paraformaldehyde and stained the nuclei with 4,6-diamidino-2-phenylindole blue. We used a SP5 confocal microscopy to detect the red signals in the ESCs.

### Masson's Trichrome staining

14 days after MD, the uteruses were fixed with 4% paraformaldehyde for 48 h before embedding in paraffin. All slices were cross sections of the uteruses and were stained with Hematoxylin, Aniline Blue and Acid Magenta. We observed these slices under a microscope with 400× magnification. We calculated the fibrotic areas using HistoQuest tissue analysis software.

### Histological section analysis

We collected the damaged uteruses, fixed them with 4% concentration of paraformaldehyde overnight, dehydrated them in stratified alcohols, and then embedded them in paraffin. We consecutively sliced the embedded samples into sections of 5 mm thickness and normally stained them with HE. We counted gland numbers and averaged five randomly selected high-power fields (HPF) taken from every slide to determine the abundance of gland in uterine. We examined the thickness and the morphology of endometrium under a light microscope.

### Construction of dsRed-CD63-expressing BMSCs and detection of the exosomes secretion and uptake

We cultured BMSCs in six-well plates to 70–80% confluence, then transfected them with 5 μg/well of purified pCT-CD63-dsRed (Origene) using lipofectamine 2000 (Invitrogen) reagent, according to the manufacturer's protocols. We selected cells with 10 μg/ml puromycin dihydrochloride (Invitrogen) for 3 weeks. We further isolated the puromycin resistant and dsRed positive cells, expanded and maintained them in the selection medium. We verified the expression of dsRed-CD63 in the selected cell lines, referred to as dsRed-CD63-BMSCs by immunoblotting and immunofluorescence. We administered the rats (*n*=4) subjected to MD with these dsRed-CD63-BMSCs at 24 h post injury. At 14 days after MD, rats were deeply anesthetized with 3% mebumalnatrium (3.9 ml/kg). We snap froze the rat uteruses in liquid nitrogen and kept them at −80°C. We generated 8-μm-thick frozen uterus sections as noted above, and employed immunofluorescent staining to distinguish ESCs with vimentin (1:200, ab137321, Abcam) followed with corresponding GFP-conjugated secondary antibodies. We employed a laser scanning confocal microscope to examine the secretion of exosomes from dsRed tagged BMSCs and the uptake of exosomes by ESCs.

### 3′-UTR luciferase reporter analyses

We performed the 3′-UTR luciferase reporter assays as previously reported (Grens and Scheffler, 1990). We seeded the ESCs at 3×10^5^ cells/well in six-well plates 24 h prior to transfection with 0.5 mg/ml of PRL reporter plasmids (Origene), CMV-galactosidase construct (Origene) and miRNA inhibitors (Origene) using Lipofectamine2000 (Invitrogen) in OptiMEM medium (Sigma). 24 h later, we seeded the engineered BMSCs containing miR-340 or NTC on the top of a transwell insert. We harvested the ESCs 72 h post transfection and performed the luciferase and pCMV assays with the Dual-Luciferase reporter assay system (Promega) according to the manufacturer's methods. The 3′-UTR of rat TGF-βR1 gene contains an 8-mer (25–32: CUUUAUAA) miR340 binding site conserved across multiple species.

### Statistical analysis

The data were represented as mean±standard deviation (s.d.). We utilized one-way analysis of variance (ANOVA) to determine differences among multiple groups. We performed Dunnett's tests to decide the differences between pairs. We used repeated measures analysis to evaluate histological measurement on multiple regions per subject. *P*<0.05 was advised to have significant difference (*P<0.05 or **P<0.01).
